# Human cancer cell line microRNAs associated with *in vitro* sensitivity to paclitaxel

**DOI:** 10.3892/or.2013.2847

**Published:** 2013-11-13

**Authors:** NING CHEN, HYE SOOK CHON, YIN XIONG, DOUGLAS C. MARCHION, PATRICIA L. JUDSON, ARDESHIR HAKAM, JESUS GONZALEZ-BOSQUET, JENNIFER PERMUTH-WEY, ROBERT M. WENHAM, SACHIN M. APTE, JIN Q. CHENG, THOMAS A. SELLERS, JOHNATHAN M. LANCASTER

**Affiliations:** 1 Department of Women’s Oncology, H. Lee Moffitt Cancer Center and Research Institute, Tampa, FL 33612, USA; 2 Experimental Therapeutics Program, H. Lee Moffitt Cancer Center and Research Institute, Tampa, FL 33612, USA; 3 Department of Anatomic Pathology, H. Lee Moffitt Cancer Center and Research Institute, Tampa, FL 33612, USA; 4 Department of Cancer Epidemiology, H. Lee Moffitt Cancer Center and Research Institute, Tampa, FL 33612, USA; 5 Department of Molecular Oncology, H. Lee Moffitt Cancer Center and Research Institute, Tampa, FL 33612, USA; 6 Department of Oncologic Sciences, H. Lee Moffitt Cancer Center and Research Institute, Tampa, FL 33612, USA

**Keywords:** cancer, chemosensitivity, microRNA, NCI60

## Abstract

Paclitaxel is a mainstay of treatment for many solid tumors, and frequently, clinical outcome is influenced by paclitaxel sensitivity. Despite this, our understanding of the molecular basis of paclitaxel response is incomplete. Recently, it has been shown that microRNAs (miRNAs) influence messenger RNA (mRNA) transcriptional control and can contribute to human carcinogenesis. In the present study, our objective was to identify miRNAs associated with cancer cell line response to paclitaxel and to evaluate these miRNAs as therapeutic targets to increase paclitaxel sensitivity. We measured the expression of 335 unique miRNAs in 40 human cancer cell lines selected from the NCI panel. We then integrated miRNA expression data with publicly available paclitaxel-sensitivity (GI_50_) data for each of the 40 cell lines to identify miRNAs associated with paclitaxel sensitivity. Ovarian cancer cell lines with differential miRNA expression and paclitaxel sensitivity were transiently transfected with miRNA precursors and inhibitors, and the effects on *in vitro* cell paclitaxel sensitivity were evaluated. Pearson’s correlation identified 2 miRNAs (miR-367 and miR-30a-5p) associated with the NCI40 cell line *in vitro* paclitaxel response (P<0.0003). Ovarian cancer cells were selected based on the association between paclitaxel sensitivity and miR-367/miR-30a-5p expression. Overexpression of miR-367 in the paclitaxel-sensitive cells [PA1; IC_50_, 1.69 nM, high miR-367 (2.997), low miR-30a-5p (−0.323)] further increased paclitaxel sensitivity, whereas miR-367 depletion decreased paclitaxel sensitivity. In contrast, overexpression and depletion of miR-30a-5p in the paclitaxel-resistant cells [OVCAR4; IC_50_, 17.8 nM, low miR-367 (−0.640), high miR-30a-5p (3.270)] decreased and increased paclitaxel sensitivity, respectively. We identified and successfully targeted miRNAs associated with human cancer cell line response to paclitaxel. Our strategy of integrating *in vitro* miRNA expression and drug sensitivity data may not only aid in the characterization of determinants of drug response but also in the identification of novel therapeutic targets to increase activity of existing therapeutics.

## Introduction

Paclitaxel is a plant alkaloid that was developed from the bark of the Pacific yew tree, *Taxus brevifolia*(1). Paclitaxel is a taxane that stabilizes and disrupts microtubules required for cell division, resulting in cell death (2,3). Despite extensive clinical use in the treatment of patients with lung, ovarian and breast cancer, long-term survival is frequently compromised by the development of paclitaxel resistance, for which the molecular basis remains to be fully delineated. microRNAs (miRNAs) are non-coding, 21–25 nucleotide regulatory RNAs that affect the stability and/or translational efficiency of messenger RNA (mRNAs) (4). Thousands of miRNAs are predicted to exist in the human genome (5) of which 1,100 human miRNAs have been identified, collectively targeting more than 19,000 human genes (www.microRNA.org). Deregulation of miRNAs has been implicated in the development of many types of human cancers (6,7), suggesting that some miRNAs function as oncogenes or tumor suppressors (8,9). It has been reported that loss of *let7* may influence the development of lung cancer as it negatively regulates let60/RAS (10), whereas miRs-34a-c may play an important role in the tumor-suppressor function of p53 (11,12) and miR-181a was found to be related to a morphological subclass of acute myeloid leukemia (13). Some studies have suggested that miRNAs may also influence chemosensitivity (14–16). It has been shown that miR-221/222 overexpression reduces p27^Kip1^ levels and induces tamoxifen resistance due to cell cycle inhibition (17), whereas inhibition of miR-21 increases apoptosis in lung adenocarcinoma epithelial cell line A549 after NSC 265450 (nogamycin) and NSC 670550 treatment by downregulating Bcl2 protein (14).

In the present study, we integrated miRNA data for lung, colon, breast, ovarian, kidney, skin (melanoma), prostate, central nervous system (CNS), and hematologic (leukemia) cancer cell lines with GI_50_ paclitaxel-sensitivity data in an effort to identify miRNAs associated with paclitaxel response. Furthermore, we evaluated the effect of *in vitro* targeted modulation of these miRNAs on paclitaxel sensitivity.

## Materials and methods

### Cell culture and reagents

A subset of 40 of the NCI60 cancer cell line panel was obtained from the National Cancer Institute (NCI) Developmental Therapeutics Program (Table I). Ovarian cancer (OVCA) cell lines in addition to those on the NCI60 panel were obtained from the American Type Culture Collection (ATCC, Manassas, VA, USA; CAOV3, OV90, OVCAR3 and SKOV3), the European Collection of Cell Cultures (Salisbury, UK; A2780CP and A2780S), Kyoto University (Kyoto, Japan; M41, M41CSR, Tyknu, and TyknuCisR), or as kind gifts from Dr Patricia Kruk, Department of Pathology, College of Medicine, University of South Florida, Tampa, FL, and Susan Murphy, Department of OBGYN/Division of Gynecologic Oncology, Duke University, Durham, NC (HeyA8, IGR-OV1, IMCC3, IMCC5, MCAS, OVCA420, OVCA429, OVCA432, OVCA433, FUOV1, PA1, PEO1, PEO4, T8, TOV-112D, TOV-21-G, Dov13, OVCAR10, OVCAR8, OVCAR5, OVCAR4 and OVCAR2). Human stem cell lines (H9) were obtained from WiCell (Madison, WI, USA). All cancer cell lines were cultured in RPMI-1640 medium supplemented with 1% non-essential amino acids, 1% sodium pyruvate and 10% fetal bovine serum. H9 cells were cultured according to the manufacturer’s protocol. Cells were cultured for 2–3 passages before experimentation. Paclitaxel was obtained from Sequoia Research Products Ltd., (Oxfordshire, UK), dissolved in DMSO at a concentration of 100 mM and stored at −20°C.

### miRNA extraction and expression profiling

Total RNA was extracted from 1×10^6^ log-phase cells using the mirVana miRNA isolation kit (Life Technologies) according to the manufacturer’s instructions. The yield and quality of total RNA for each cell line were determined using an Agilent Bioanalyzer. The RNA integrity number (RIN) of all samples was in a range of 8–10 and no genomic DNA contamination was detected. Total RNA (10 μg) from each cell line was then subjected to miRNA expression profiling. The cell line samples were co-hybridized to printed arrays that contained a 562 Ambion mirVana miRNA probe set (Ambion, Austin TX, USA) and 632 of Invitrogen’s NCode Multi-Species miRNA probes (Invitrogen, Carlsbad, CA, USA), which contain >800 unique human miRNAs. The hybridized arrays were scanned on a GenePix 4000B scanner, and expression data were generated using the GenePix Pro software (Molecular Devices, Sunnyvale, CA, USA).

### Transfection of miRNA

Cell lines were transfected with pre-miR-367, pre-miR-30a-5p, anti-miR-367 or anti-miR-30a-5p (Life Technologies) using siPORT NeoFX transfection reagent, according to the manufacturer’s protocol. The concentrations of miRNA and the transfection reagent were optimized by the manufacturer’s recommendations prior to experimentation. Log-phase cells (~60% confluence) were incubated with 6.25 μM precursor or inhibitor miRNAs in the presence of 50 nM transfection reagent for 4 h. pre-miR negative control 1 precursor and anti-miR negative control 1 were used as controls for miRNA overexpression and depletion experiments, respectively. Control miRNAs were transfected under the same condition as the experimental miRNAs. Both control miRNAs have mechanisms similar to the pre-miRNA precursors and anti-miRNA inhibitors but have no observed effects on known miRNA functions.

### Comparative C_T_ RT-PCR

Real-time comparative C_T_ RT-PCR was used to determine relative miRNA levels. miRNAs were converted into cDNA using miRNA sequence-specific primers and the TaqMan^®^ microRNA reverse transcription (Applied Biosystems). Total RNA (10 ng) was used for each 15-μl reverse transcription reaction. Comparative C_T_ RT-PCR was performed on the Applied Biosystems StepOne Real-Time PCR system, according to the manufacturer’s protocols. Briefly, 1.33 μl of miRNA-specific cDNA was combined with sequence-specific TaqMan miRNA assays and TaqMan Universal PCR Master Mix, no AmpErase UNG in a total reaction volume of 20 μl. RNU44 was used as the endogenous miRNA control for normalization. RNU44 was considered a qualified candidate of endogenous control based on preliminary experimentation (data not shown). miRNA expression levels were analyzed using StepOne Real-Time PCR instrument software.

### Growth inhibition assay

Cells were seeded in 96-well plates (Perkin-Elmer) at a density of 6×10^4^ cells/ml and incubated overnight at 37°C. Cells were incubated with the indicated concentrations of paclitaxel for 72 h, and cell viability was assessed using the CellTiter-Glo™ luminescent cell viability assay kit (MTS kit; Promega). Luminescence was recorded using Wallace Victor2™ 1420 Multilabel Counter (Perkin-Elmer). Wells containing medium without cells were used to obtain background luminescence. All experimental wells and controls were set up in triplet. Paclitaxel log_10_ (IC_50_) values for transfected cell lines were calculated using the sigmoidal dose-response (variable slope) curve equation (Prism 5). Dose-curve graphs were generated using GraphPad (Prism 5). Cells cultured with transfected medium without miRNA precursors or inhibitors were used as controls. The effects of increased pre-miR and anti-miR levels due to transfection on cell viability were assessed 48 h post-transfection using MTS assays.

### Statistical analysis

Two color spotted array data for miRNA levels were generated from the GenePix Pro software. Background subtraction (18) and Loess normalization (19) were performed using Limma software. Replicate probe sets were averaged by their design (Ambion or Invitrogen). Processed data were then analyzed using SAM (significance analysis of microarrays) software (20). Missing values were imputed with SAM’s nearest neighbor imputer, where *k* was set to 10. For each drug, Pearson’s correlation test was used to identify those miRNAs with expression values associated with sensitivity measured by GI_50_.

### Pathway analysis

The miRanda database was used to identify the mRNA targets of miRNAs found to be associated with *in vitro* sensitivity to chemotherapy. The identified mRNA targets were subjected to GeneGo MetaCore analysis to determine biological signaling pathway representation. P<0.05 represented statistical significance of the association between the mRNA targets of the miRNAs and the biological pathways.

## Results

### Correlation of miRNA expression and paclitaxel sensitivity/resistance

Paclitaxel sensitivity (GI_50_) data for the subset of 40 cancer cell lines of the NCI60 cell panel (3 leukemia, 6 melanoma, 8 non-small cell lung, 6 colon, 4 central nervous system, 2 ovarian, 7 renal, 2 prostate and 2 breast cancer cell lines) was obtained from NCI Website (http://dtp.nci.nih.gov/dtpstandard/cancerscreeningdata/index.jsp). Based on miRNA expression and GI_50_ data, Pearson’s correlation test identified 35 miRNAs associated with *in vitro* paclitaxel sensitivity (P<0.05). miR-367 and miR-30a-5p demonstrated the highest level of statistical significance in association with sensitivity to paclitaxel (P<0.0003) (Table II).

### Selection of cell lines

**Figure 1:**
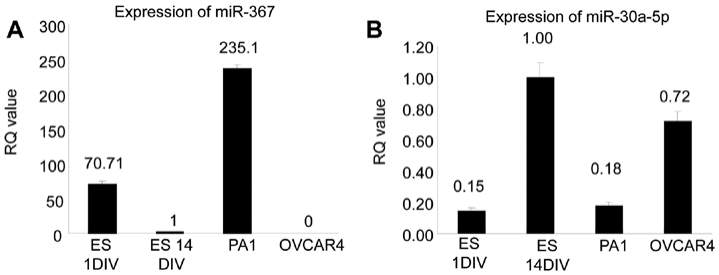
Differential expression of miR-367 and miR-30a-5p by quantitative RT-PCR. The differential expression of (A) miR-367 and (B) miR-30a-5p was confirmed by RT-PCR in the ovarian cancer cell lines, PA1 and OVCAR4. Embryonic stem cells cultured for 1 day (early-stage, ES1DIV) and 14 days (late-stage, ES14DIV) were used as controls for positive and negative miRNA expression, respectively. RNU44 was used as endogenous control. ES14DIV was used as the sample reference control. The value of the reference control was set as 1.

The OVCA cell lines PA1 and OVCAR4 were selected for further evaluation based on associations between paclitaxel sensitivity and miR-367/miR-30a-5p expression. An evaluation of the OVCA cell lines showed PA1 to have a higher relative expression value of miR-367 (2.997), a lower relative expression value of miR-30a-5p (−0.32), and relative sensitivity to paclitaxel-induced cell growth arrest (IC_50_, 1.69 nM). In contrast, OVCAR4 showed a lower relative expression value of miR-367 (−0.64), a higher relative expression values of miR-30a-5p (3.27), and was more resistant to paclitaxel (IC_50_, 17.8 nM). The differential expression of miR-367 and miR-30a-5p in PA1 and OVCAR4 cells was confirmed by quantitative RT-PCR (Fig. 1). As a reference, miRNA expression was compared to embryonic stem cells, which reportedly express miR-367 in the early stage and reduced miR-367 expression in the late stage (21). Embryonic stems cells were defined as early vs. late stage based on culture duration of 1 (ES1DIV) vs. 14 (ES14DIV) days, respectively. As shown in Fig. 1A, using ES14DIV as the reference [relative expression (RQ)=1], the relative expression (RQ value) of miR-367 PA1 cells was 235.1, compared to RQ=70.71 for ES1DIV (positive control) and RQ=0 for OVCAR4 cells. In contrast, normalized to ES14DIV (RQ=1), OVCAR4 had the highest expression of miR-30a-5p with an RQ=0.72, compared to RQ=0.18 for PA1 cells (Fig. 1B).

### Evaluation of miR-367 and miR-30a-5p as therapeutic targets

To determine the value of miR-367 and miR-30a-5p as therapeutic targets in OVCA cells, miR-367 was overexpressed or depleted in the paclitaxel-resistant cell line PA1 (high miR-367, low miR-30a-5p), through transient transfection of the miRNA precursor (pre-miR-367) and inhibitor (anti-miR-367), respectively. In contrast, the paclitaxel-sensitive OVCA cell line OVCAR4 (low miR-367, high miR-30a-5p) was transfected with the miR-30a-5p precursor (pre-miR-30a-5p) and inhibitor (anti-miR-30a-5p). Forty-eight hours after transfection, cells were incubated with increasing doses of paclitaxel for 72 h and evaluated for cell viability using the CellTiter-Glo™ luminescent assay.

**Figure 2:**
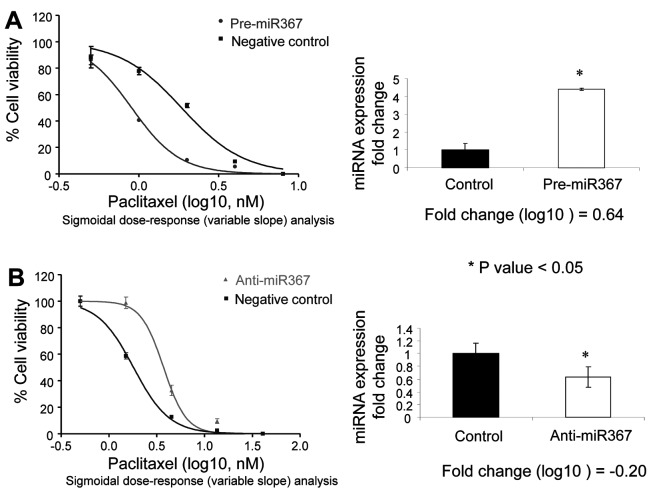
Modulation of miRNA expression affects paclitaxel sensitivity. PA1 cells (high miR-367 expression, low miR-30a-5p expression) were evaluated for paclitaxel-induced growth arrest at 72 and 48 h after transient transfection of (A) pre-miR-367 precursor miRNA and (B) anti-miR-367 inhibitor miRNA. Changes in miRNA levels were evaluated 48 h after transfection by comparative C_T_ RT-PCR. The negative control was set as the reference sample and the fold-change as 1. The endogenous control was RNU44. The fold-change before and after transfection was calculated by 2^−ΔΔCt^.

Compared to the mock transfection controls, PA1 cells transfected with pre-miR-367 showed a 57% decrease in cell survivability 48 h after transfection, whereas transfection of anti-miR-367 did not affect cell survival. Transfection of pre-miR-30a-5p and anti-miR-30a-5p had no effect on the survivability of PA1 cells (data not shown). Despite the decrease in cell survival, transfection of pre-miR-367 in PA1 cells resulted in a 0.64 log_10_-fold-change in miR-367 expression and an increase in paclitaxel sensitivity when compared to the precursor negative control (Fig. 2A). In contrast, transfection of PA1 cells with anti-miR-367 resulted in a −0.2 log_10_-fold-change and a decrease in paclitaxel sensitivity (Fig. 2B).

**Figure 3:**
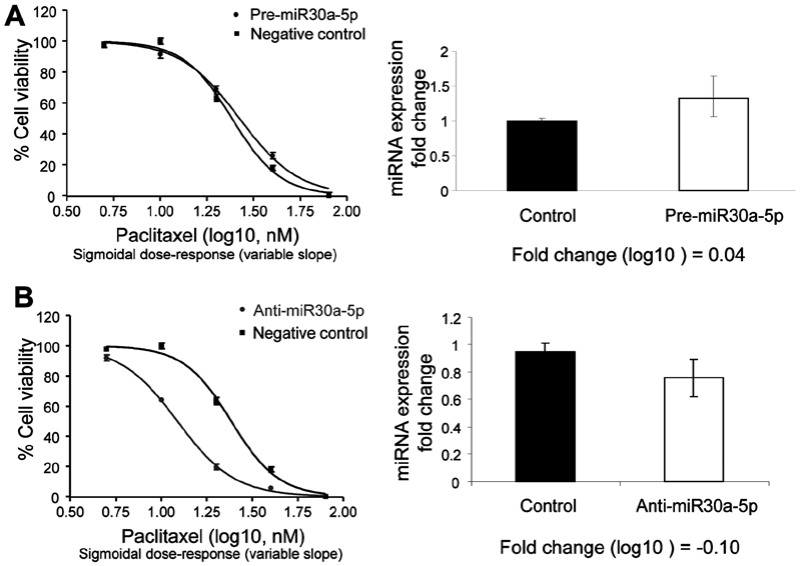
Modulation of miRNA expression affects paclitaxel sensitivity. OVCAR4 cells (low miR-367 expression, high miR-30a-5p expression) were evaluated for paclitaxel-induced growth arrest at 72 and 48 h after transient transfection of (A) pre-miR-30a-5p precursor miRNA and (B) anti-miR-30a-5p inhibitor miRNA. Non-targeting pre-miRNA and anti-miRNA negative controls were used as references. Changes in miRNA levels were evaluated 48 h after transfection by comparative C_T_ RT-PCR. The negative control was set as the reference sample and the fold-change as 1. The endogenous control was RNU44. The fold-change before and after transfection was calculated by 2^−ΔΔCt^.

In the intrinsically paclitaxel-resistant cell line (OVCAR4), which has high relative expression of miR-30a-5p and low relative expression of miR-367, overexpression of the miR-30a-5p precursor (0.04 log_10_-fold change) slightly decreased paclitaxel-induced sensitivity, whereas depletion of miR-30a-5p (−0.10 log_10_-fold change) increased paclitaxel-induced growth arrest (Fig. 3).

### Pathways involved in the deregulation of miRNAs

To evaluate the potential influence of miR-367 and miR-30-5p on various cellular processes, we identified the predicted mRNA target genes of these miRNAs using the miRanda database (22). This database hosts target sites for 1,100 human miRNAs and 16,228,619 predicted miRNA targets in 34,911 distinct 3′UTRs of 19,898 human genes. The miRanda database predicted 1,536 and 2,320 target genes associated with miR-367 and miR-30a-5p, respectively (mirSVR score >−0.15, mirSVR score ranged from −0.1 to −1.35) (20). These predicted miRNA targets were further analyzed for biologic signaling pathway representation using GeneGo MetaCore software. Pathway modeling identified 16 pathways represented among the predicted target genes for miR-367 (P<0.0001) and 20 pathways were represented among the target genes for miR-30a (P<0.0001) (Table III).

## Discussion

The efficacy of cancer treatment is frequently limited by intrinsic and acquired resistance to chemotherapy. Despite progress in delineating the molecular determinants of cancer chemo-response, a comprehensive understanding of the factors that underlie drug resistance remains elusive.

Evidence is accumulating to support a role for miRNAs in the development and progression of human cancer (7,23,24). Moreover, recent data also suggest that miRNAs may influence cancer cell response to chemotherapy (14,16,25) by mechanisms that may be both cancer cell-type or drug specific. Previous studies have shown that paclitaxel sensitivity may be associated with the expression of miR-200c in both ovarian (26) and gastric cancer (27), miR-148 in prostate cancer cells (28), miR-337, miR-34 and miR-135a in lung cancer (29–31), miR-22 in colon cancer (32), and miR-125b and miR-21 in breast cancer (33,34).

In the present study, miRNA expression data integrated with publicly available chemosensitivity data for 40 human cancer cell lines (representing 9 different cancer cell types) identified 35 miRNAs to be associated with *in vitro* paclitaxel sensitivity (P<0.05). Two of these miRNAs, miR-367 and miR-30a-5p, were selected for further experimentation based on associations between paclitaxel sensitivity and miR-367/miR-30a-5p expression. The effects of miR-367 and miR-30a-5p expression on chemosensitivity were investigated in OVCA cell lines shown to have differential expression of these miRNAs. The OVCA cell line PA1 was found to be relatively sensitive to paclitaxel-induced cell death and have relatively high expression of miR-367 and low expression of miR-30a-5p. In contrast, OVCAR4 cells were found to have almost no expression of miR-367 and relatively high expression of miR-30a-5p and were relatively resistant to paclitaxel. In PA1 cells, the overexpression and depletion of miR-367 increased the sensitivity and resistance of these cell lines to paclitaxel-induced growth arrest and cell death, respectively. In contrast, in OVCAR4 cells, an increase in miR-30a-5p expression was associated with decreased paclitaxel sensitivity, whereas a depletion of miR-30a-5p was associated with an increase in paclitaxel sensitivity. The mechanism by which these miRNAs affect chemosensitivity was not determined. However, miR-367, which belongs to the miR302 cluster, is only present in embryonic stem cells and is significantly decreased after cells differentiate (21). miR-302 and miR-367 not only participate in the processes of maintaining cell self-renewal and pluripotency in embryonic stem cells but are also overexpressed in various cancer cells (21,35–38) and may play a role in chemosensitivity (39). Similarly, miR-30a-5p has been reported to be differentially expressed in various malignancies, including lung, thyroid, anaplastic and gastric cancer (40–44) and has been associated with survival of patients with cancer (45,46).

Bioinformatic analyses of the predicted miR-367 target genes indicated that miR-367 may have an important regulatory role in the expression or activity of 16 biologic signaling pathways. Notably, the majority of these miR-367-influenced pathways, such as signal transduction/PKA signaling, signal transduction/AKT signaling, cAMP signaling, and apoptosis and survival/BAD phosphorylation, influence cell survival through regulating cell cycle and cell apoptosis and maintaining cell self-renewal and stemness (Table III), although the role of miR-367 in carcinogenesis and chemosensitivity is largely unknown.

miR-30a-5p was predicted to influence the expression of 20 biologic signaling pathways, several of which are also known to influence cellular survival, such as apoptosis and survival/FAS signaling cascades and apoptosis and survival/caspase cascade. However, the majority of pathways under the influence of miR-30a-5p’s appears to involve cytoskeletal remodeling and cell migration (Table III).

The present study demonstrated that the integration of miRNA expression data with existing chemosensitivity data from the NCI40 cell line set may provide insight into miRNAs that influence *in vitro* paclitaxel sensitivity. However, it should be acknowledged that such an approach may preferentially identify those miRNAs that were influential in determination of chemosensitivity across tumor types and may not identify those miRNAs that have a cancer-specific influence on the response to paclitaxel. Although data such as these provide an important contribution to our knowledge of the underpinnings of cancer cell response to therapeutic agents, it should be recognized that a comprehensive understanding of the biologic determinants of chemo-response will ultimately require us to incorporate information on additional variables such as DNA sequence and copy number, mRNA expression (vs. predicted mRNA targets), and protein levels and post-translational modifications. Our data contribute to the growing body of evidence suggesting that miRNAs have potential utility as personalized medicine biomarkers of cancer cell response to therapy and, moreover, may also represent viable therapeutic targets to increase cancer cell chemosensitivity.

## Figures and Tables

**Table I:** Cancer cell lines subjected to miRNA expression analyses.

Origin of cancer tissue	Cell lines
Lung	NCI-H460, NCI-H522, NCI-H322M, HOP62, A549, EKVX, MALME-3M, NCI-H226
Colon	HT29, HCT-116, SE-620, HCT-15, HCC2998, COLO205
Breast	HS-578T, NCI/ADR-RES
Ovarian	OVCAR8, OVCAR4
Renal	ACHN, SN-12C, 786-O, CAKI-1, UO-31, TK-10, A498
Melanoma	SK-MEL-28, UACC-257, M14, UACC-62, SK-MEL-2, LOX-IMVI
Prostate	DU-145, PC-3
CNS	SF-295, SF-539, SNB-75, U251
Leukemia	HL-60, RPMI8226, K562

**Table II:** microRNAs associated with paclitaxel sensitivity.

miRNA	P-value	Up/down
hsa_miR_367	0.00022	Down
hsa_miR_30a_5p	0.00028	Up
hsa_miR_141	0.00115	Down
hsa_miR_30a_3p	0.00306	Up
hsa_miR_516_3p	0.00318	Up
hsa_miR_377	0.00350	Down
hsa_miR_134	0.00371	Up
hsa_miR_142_5p	0.00767	Down
hsa_let_7e	0.01000	Up
hsa_miR_29c	0.01017	Down
hsa_miR_218	0.01105	Down
hsa_miR_17_3p	0.01123	Down
hsa_miR_17_5p	0.01288	Down
hsa_miR_130a	0.01289	Up
hsa_miR_195	0.01939	Down
hsa_miR_99b	0.02003	Up
hsa_miR_338	0.02508	Up
hsa_miR_106a	0.02611	Down
hsa_miR_193b	0.02720	Up
hsa_miR_515_3p	0.02884	Up
hsa_miR_374	0.02889	Up
hsa_miR_125a	0.02993	Up
hsa_miR_192	0.03023	Down
hsa_miR_30c	0.03039	Up
hsa_miR_95	0.03684	Down
hsa_miR_452_AS	0.03703	Down
hsa_miR_489	0.03774	Up
hsa_miR_32	0.03897	Down
hsa_miR_373*	0.03938	Up
hsa_miR_130b	0.04136	Down
hsa_miR_19b	0.04385	Down
hsa_miR_126	0.04543	Down
hsa_miR_148a	0.04650	Down
hsa_miR_376b	0.04835	Up
hsa_miR_7	0.04835	Up

Positive correlation, up; negative correlation, down.

**Table III:** miR-367/miR-30a-5p target gene-involved pathways (P<0.0001).

	P-value	Objects/networks
miR-367 target gene-involved pathways (P<0.0001)		
Signal transduction_PKA signaling	1.84E-07	14/15
Signal transduction_cAMP signaling	2.58E-07	12/38
Development_Thrombopoietin-regulated cell processes	2.63E-07	13/45
Development_A2A receptor signaling	1.16E-06	12/43
Immune response_IL-23 signaling pathway	2.44E-06	9/25
Development_TGF-β-dependent induction of EMT via RhoA, PI3K and ILK	2.54E-06	12/46
Signal transduction_Activation of PKC via G-Protein coupled receptor	1.01E-05	12/52
Neurophysiological process_Glutamate regulation of Dopamine D1A receptor signaling	1.32E-05	11/45
Development_Role of HDAC and calcium/calmodulin-dependent kinase (CaMK)	1.53E-05	12/54
in control of skeletal myogenesis		
Development_PACAP signaling in neural cells	2.09E-05	10/39
Translation_Insulin regulation of translation	4.19E-05	10/42
Apoptosis and survival_BAD phosphorylation	4.19E-05	10/42
Development_IGF-1 receptor signaling	4.68E-05	11/51
Signal transduction_AKT signaling	5.22E-05	10/43
Transport_Clathrin-coated vesicle cycle	6.08E-05	13/71
Neurophysiological process_ACM regulation of nerve impulse	9.64E-05	10/46
miR30a-5p target gene-involved pathways (P<0.0001)		
Cytoskeleton remodeling_TGF, WNT and cytoskeletal remodeling	8.72E-08	25/111
Cytoskeleton remodeling_Cytoskeleton remodeling	2.81E-07	23/102
Cell adhesion_Ephrin signaling	1.02E-06	14/45
Development_Thrombopoietin-regulated cell processes	1.02E-06	14/45
Development_HGF signaling pathway	1.84E-06	14/47
Development_WNT signaling pathway. Part 2	8.82E-06	14/53
Muscle contraction_Regulation of eNOS activity in endothelial cells	2.05E-05	15/64
Development_Regulation of epithelial-to-mesenchymal transition (EMT)	2.05E-05	15/64
Apoptosis and survival_FAS signaling cascades	2.12E-05	12/43
Development_Membrane-bound ESR1: interaction with growth factor signaling	2.12E-05	12/43
Cardiac hypertrophy_NF-AT signaling in cardiac hypertrophy	2.5E-05	15/65
Immune response_ETV3 affect on CSF1-promoted macrophage differentiation	2.58E-05	10/31
Development_Role of IL-8 in angiogenesis	2.7E-05	14/58
Development_Ligand-independent activation of ESR1 and ESR2	2.73E-05	12/44
Cell adhesion_Chemokines and adhesion	4.13E-05	19/100
Translation_Regulation of EIF4F activity	4.36E-05	13/53
Apoptosis and survival_Caspase cascade	4.75E-05	10/33
Development_PIP3 signaling in cardiac myocytes	5.59E-05	12/47
PGE2 pathways in cancer	6.61E-05	13/55
DNA damage_Role of SUMO in p53 regulation	7.41E-05	7/17
